# A Combination of Serum Biomarkers in Elderly Patients with Sarcopenia: A Cross-Sectional Observational Study

**DOI:** 10.1155/2022/4026940

**Published:** 2022-02-21

**Authors:** Lin Ying, Qin Zhang, Yun-mei Yang, Jian-ying Zhou

**Affiliations:** ^1^Department of Geriatrics, The First Affiliated Hospital, School of Medicine, Zhejiang University, Hangzhou 310003, China; ^2^Key Laboratory of Diagnosis and Treatment of Aging and Physic-Chemical Injury Diseases of Zhejiang Province, The First Affiliated Hospital, School of Medicine, Zhejiang University, Hangzhou 310003, China; ^3^Department of Respiratory Disease, Thoracic Disease Center, The First Affiliated Hospital, School of Medicine, Zhejiang University, Hangzhou 310003, China

## Abstract

**Background:**

The pathogenesis of sarcopenia in the elderly has not yet been fully understood. This study aimed to explore the relationship between sarcopenia and several serum biomarkers in elderly population.

**Methods:**

It was an observational cross‐sectional study of data collected from 70 patients. According to the criteria of the Asian Working Group for Sarcopenia (AWGS), subjects were divided into the sarcopenia group and nonsarcopenic group. We compared age, body mass index (BMI), biochemical indexes, smoking status, underlying disease, muscle mass, handgrip strength (HS), gait speed (GS), skinfold thickness, muscle thickness, and IL-6, IL-10, IL-17A, and TNF-*α* levels between these groups.

**Results:**

Of the 70 subjects, 35 patients were diagnosed with sarcopenia. The number of men was higher than that of women in both groups. The patients with sarcopenia were older and had lower BMI and muscle thickness but higher SARC-F questionnaire scores. However, the difference in smoking status and skinfold thickness between these two groups were not statistically significant. Higher IL-6, IL-17A, and TNF-*α* levels were observed in participants with sarcopenia (*P* < 0.05). Patients with sarcopenia had a lower IL-10 level. Positive associations were present between the severity of sarcopenia and IL-6, IL-17A, and TNF-*α* levels, while there was an inverse correlation between the presence of sarcopenia and IL-10 level.

**Conclusions:**

Our research found that in sarcopenic elderly subjects, the serum levels of several biomarkers, such as IL-6, IL-17A, and TNF-*α*, were higher than those in nonsarcopenic elderly persons. Further studies are needed to explore the possible molecular mechanisms and discover new therapeutic targets.

## 1. Background

The rapid increase in the elderly population represents a global health problem [1], together with the increased incidence of age-related diseases, such as osteoporosis and sarcopenia [2]. It has been reported that 0.5–1.0% of skeletal muscle mass is lost per year beyond the age of 30 years [3]. Sarcopenia, defined as the progressive loss of skeletal muscle mass, is associated with a reduction in the number and size of muscle fibers, leading to a decrease in muscular strength and endurance [4]. It may increase the risk of adverse outcomes, such as falls, disability, fractures, hospitalization, and mortality [5, 6].

Nowadays, as more attention is paid to sarcopenia in the last 10 years, significant progress has been made in basic and clinical research. However, the mechanisms underlying the etiology and progression of sarcopenia still remain poorly understood. Studies suggest that multiple mechanisms underlie the development of sarcopenia [7, 8], including impaired insulin signaling, imbalanced anabolic and catabolic energy metabolism, immune dysfunction, systemic chronic inflammation, and increased oxidative stress [9].

Inflammation is considered to serve as a biomarker for accelerated aging and is one of the hallmarks of aging biology [10]. It is recognized that with age, the mechanisms regulating inflammation become impaired in a manner that might contribute to the susceptibility of elderly persons to infection and age-related chronic diseases, such as sarcopenia [11]. This dysregulation has been inferred in part from an observation of increased circulating proinflammatory cytokines, such as IL-6 and TNF-*α*, in elderly persons even in the absence of infection, suggesting a low-level chronic inflammation specific to aging known as “inflammaging” [12–14]. Furthermore, fatty tissue increase with aging. Several studies have revealed that the adipose tissue is the main source of inflammatory molecules, such as IL-6 and MCP1 [15]. Another study showed that cytokines that are produced by adipocytes may have a direct effect on physical function by accelerating the changes in body composition that are typical of the aging process, namely fat gain and loss of muscle mass [16].

So far, studies have reported that serum levels of proinflammatory cytokines, such as TNF-*α* and IL-6, were increased in elderly sarcopenia cases [17, 18]. It is known that IL-6 enhances the differentiation of Th17 cells [19–21], which are a subset of T helper cells and can secrete several potent proinflammatory cytokines, including IL-17A and IL-17F, in many inflammatory and autoimmune diseases [22, 23]. However, the role of Th17 cells and IL-17 in elderly sarcopenia patients is unknown. To investigate this, we measured serum concentrations of the inflammatory cytokines IL-6, TNF-*α*, and IL-17A and estimated the association between IL-6 and IL-17A levels in elderly individuals with sarcopenia.

## 2. Methods

### 2.1. Research Subjects

A total of 70 elderly subjects (>60 years old, 43 men and 27 women) were enrolled in this cross-sectional study. They underwent general medical examinations between January 2020 and September 2020 in the Geriatrics Department. Among these subjects, 35 patients were diagnosed with sarcopenia according to the criteria of the Asian Working Group for Sarcopenia (AWGS) [24]. The covariates were obtained from the hospital information systems: age, sex, weight, height, smoking status, and the following comorbidities: diabetes, chronic obstructive pulmonary disease (COPD), and a history of smoking were reported to be important predictors for the onset of sarcopenia [25–27]. This study was approved by the Ethics Committee of The First Affiliated Hospital, School of Medicine, Zhejiang University, in accordance with the Helsinki Declaration. Written informed consent was obtained from all subjects.

### 2.2. Inclusion Criteria

All participants were over 60 years old and could walk by themselves. According to the AWGS criteria for sarcopenia in older persons [24], diagnosis is based on the presentation of criterion 1 (low muscle mass, <7.0 kg/m^2^ for men and 5.7 kg/m^2^ for women based on bioelectrical impedance analysis (BIA)) plus either criterion 2 (low muscle strength, handgrip strength (HS) <28 kg for men and <18 kg for women) or criterion 3 (poor physical performance) with recommended cutoff values for muscle mass measurements. The criteria for low physical performance were a 6 m walk <1.0 m/s, a short physical performance battery score ≤9, or a 5-time chair stand test ≥12 s. In this study, all patients were able to walk on their own, so we mainly evaluated the physical performance based on the walking speed.

### 2.3. Exclusion Criteria

The exclusion criteria were as follows: (1) subjects with tumors or severe weakness; (2) subjects who were admitted to the hospital due to acute infection; (3) subjects with endocrine or metabolic diseases that failed to be well-controlled; (4) subjects with diseases that seriously affect their health, such as heart failure, uremia, or septic shock; and (5) autoimmune diseases.

### 2.4. Demographic Variables

Height and weight were measured routinely. Body mass index (BMI) was calculated by dividing body weight by height squared (kg/m^2^). HS was measured using a hand-muscle developer, with patients using their dominant hand (WCS-II, Beijing). Physical performance was determined by gait speed over a course of 6 m.

All participants completed the SARC-F questionnaire, which assesses 5 components, namely strength, assistance in walking, rising from a chair, climbing stairs, and falls. The score ranged from 0 to 10. Skinfold thickness (biceps brachii and quadriceps femoris) was also measured twice at each site, and the mean was used for analysis.

### 2.5. Sarcopenia Assessment

#### 2.5.1. BIA

We used a dynamometer (EH101; Camry, Zhongshan, China) to measure body composition. During the test, subjects stood upright, gripped the handle, and kept their feet naturally separated (shoulder-width apart) and their arms naturally drooped. Intermittent gripping, swinging of the arms, or squats were not allowed. Tests were performed twice, and the larger value was recorded. According to AWGS recommendations, height-adjusted skeletal muscle mass (ASMI), defined as appendicular skeletal muscle mass (ASM)/height^2^ (m^2^) was used to evaluate the muscle mass.

### 2.6. Ultrasound (US) Measurements

Skeletal muscle measurements were performed for the biceps brachii and quadriceps femoris muscles. The participants lay down in bed, relaxed, and received examination by one doctor with 12 years of experience who was blinded to this study. The examiner held the US probe (2–10 MHz, Aixplorer; Aix-en-Provence, France) close to the skin, with the beam perpendicular to the surface, then probed the medial cross-section of the muscle and found the largest cross-sectional area, designated as the muscle thickness, measured with an accuracy of 0.01 cm.

### 2.7. Cytokine Quantification

Blood samples were obtained from the antecubital vein after admission. Biochemical indicators were tested immediately, and the remaining plasma samples were stored at −80°C for later testing. The IL-6, IL-10, IL-17A, and TNF-*α* levels were determined quantitatively using a platinum enzyme-linked immunosorbent assay (eBioscience, San Diego, CA). The minimum detectable doses of IL-6, IL-10, IL-17A, and TNF-*α* were 1.56, 0.39, 0.23, and 0.5 pg/ml, respectively. Sensitivity levels were 0.92 pg/ml (IL-6), 0.05 pg/ml (IL-10), 0.01 pg/ml (IL-17A), and <0.09 pg/ml (TNF-*α*).

### 2.8. Statistical Methods

Age, BMI, HS, skinfold thickness, muscle thickness, and ASMI were independent variables. The variables were investigated using the Kolmogorov–Smirnov test to determine normal or non-normal distributions. The data that did not coincide with normal distribution were indicated with medians (interquartile range), and the Mann–Whitney *U* test was used. The data accorded with normal distribution and homogeneity of variance were expressed as mean ± standard deviation (SD) and compared by the *t*-test. The chi-square test was used to study the relationship between genders. Pearson analysis and spearman rank correlation analysis were conducted to test correlation between variables. Statistical analyses were performed using the SPSS software version 21 (SPSS, Chicago, IL). A *P* value of <0.05 was considered statistically significant.

## 3. Results

A total of 70 elderly subjects were enrolled in this study, and 35 patients were diagnosed with sarcopenia according to the AWGS criteria. The baseline characteristics of the 70 participants are listed in Table 1. The number of men was higher than that of women in both groups, namely 21 men (60%) in the sarcopenia group and 22 men (62.9%) in the nonsarcopenic group. Intuitively, the patients without sarcopenia were younger, as the average age was 70.8, whereas the average age of 35 sarcopenia subjects was 75.1 years old.

Compared to those without sarcopenia, participants with sarcopenia had lower BMI, albumin, and muscle thickness, as per ultrasonic imaging results but higher SARC-F questionnaire scores. However, the difference in skinfold thickness, smoking status, prevalence of COPD, and diabetes between these two groups was not statistically significant.

Comparisons of IL-6, IL-10, IL-17A, and TNF-*α* levels between sarcopenia and nonsarcopenia patients are summarized in Table 2. Higher IL-6, IL-17A, and TNF-*α* levels were observed in participants with sarcopenia (*P* < 0.05). Patients with sarcopenia seemed to have a lower IL-10 level. Considering that male patients had a higher proportion of smoking and smoking might affect the level of inflammation in vivo, we then compared serum markers by gender (Table 3). A positive association was found between the high level of IL-6, IL-17A, and TNF-*α* and the prevalence of sarcopenia for both men and women. In female patients, although the level of IL-10 in patients with sarcopenia was lower than that in patients with normal muscle index, there was no statistical difference, which may be related to the small sample size.

We then performed multivariate analyses to assess the relationship between sarcopenia and serum cytokines (Table 4). Positive associations were present between the severity of sarcopenia and IL-6 and IL-17A levels, whereas there was an inverse correlation between the presence of sarcopenia and the IL-10 level. Besides, our statistical analyses also revealed that the older age and lower serum albumin were correlated with sarcopenia.

## 4. Discussion

Our study performed a cross-sectional analysis of serum concentrations of IL-6, IL-10, IL-17A, and TNF-*α* in the context of sarcopenia in elderly individuals. We found that the levels of inflammatory cytokines IL-6, IL-17A, and TNF-*α* were increased in sarcopenia patients, while the IL-10 level declined. Furthermore, there were positive associations between the severity of sarcopenia and inflammatory cytokine levels. DXA and BIA are known as the gold standard for measuring muscle mass. However, it is difficult to use these techniques in community settings. In order to select an easier and more suitable method for evaluating muscle mass in a community setting, our colleagues conducted a study. They analysed the correlation between skeletal muscle mass measured by DXA and US and found that muscle thickness measured by US was an important predictor of low skeletal muscle mass [28]. In our study, the muscle thickness of patients with sarcopenia was lower than that of patients in the control group. Both studies showed that US measurements may be used as a screening technique for muscle mass in the diagnosis of sarcopenia.

There is still controversy in the literature concerning the effect of gender on sarcopenia [29]. In our study, no difference in the prevalence of sarcopenia between men and women was observed. In addition, there was no statistically significant difference between the two groups in the percentage of patients with smoking history, COPD, and diabetes. However, the statistical analysis of data may be biased as a result of a small number of cases, and further research is needed.

China has entered an aging society in which the problem of age has become a public concern. It is expected that in 2037 China's elderly population will reach 400 million. Sarcopenia is one of the main causes of disability and quality of life reduction in the elderly. Although scholars have studied this extensively in recent years, the etiology and pathogenesis are still unclear [30, 31].

We are interested in the role of inflammation in the development of sarcopenia and have read abundant literature on the subject. Up to this time, studies on humans and animal models have suggested that several cytokines exert proinflammatory activity and play a crucial role in sarcopenia. Many studies have indicated that serum levels of inflammatory factors were increased in elderly subjects with sarcopenia and closely related to the reduction of skeletal muscle mass and strength. Bian et al. found that the IL-6 and TNF-*α* serum levels in patients with sarcopenia were higher than those of patients in the control group [32]. They also showed that BMI and visceral fat area were independent risk factors for IL-6 by multiple regression analysis. Moreover, increased IL-6 levels are reported to be positively associated with disability rate and mortality [33]. Similar results were also observed in animal studies. Gomez et al. evaluated proinflammatory cytokine production by splenic macrophages obtained from young and aged mice. They then found that splenic stromal cells from aged mice produce higher levels of IL-6 compared to young mice [34]. Except for the difference in proinflammatory cytokine secretion levels, aged IL-6 knockout mice had improved survival when compared to aged wild-type mice following the inflammatory challenge [35]. Taken together, these observations suggest inflammatory reaction has an essential role in the systemic innate immune responses of the aging process.

IL-6 is an essential cytokine to initiate the transcriptional program of Th17 cells. In the presence of TGF-*β*, IL-6 upregulates the expression of the critical Th17 transcription factor RORC (ROR*γ*t in mice) through the JAK/STAT3 pathway [36, 37]. In consideration of this role of IL-6, we hypothesized that IL-17 level may be elevated in patients with sarcopenia, and our results confirmed this hypothesis.

Except for chronic inflammation, oxidative stress has been proposed to play a role in the development of sarcopenia. Reactive oxygen species (ROS) are radical and nonradical byproducts of cellular respiration. Skeletal muscle, an excitable tissue with a high energetic demand, is widely described as a significant generator of ROS. According to Harmanis et al.'s research, the aging process is directly related to systemic oxidative stress [38]. There are two main reasons resulting in the oxidative stress situation of elderly persons, namely an accumulation of products derived from the oxidation of biological structures and a decrease in the availability of nutritional molecular antioxidants [39, 40].

In fact, increased oxidative stress and chronic inflammation have been demonstrated to occur concomitantly in the process of sarcopenia [41]. Oxidative stress can induce chronic low-grade inflammation, which has been shown to be harmful to the skeletal muscle in humans [39] as well as in animal models [42]. ROS and TNF-*α* also activate NF-*κ*B. With direct muscle injections of either cytokines (TNF-*α* and IFN-*γ*) or cancer cells as well as denervation of the sciatic nerve to induce muscle wasting in mice, researchers have shown that NF-*κ*B levels are strongly upregulated [43–45].

Even though the prevalence of sarcopenia is high, there are no specific drugs for treatment, and the muscle remains an undermedicated organ [46]. A previous study reviewed 10 pharmacological interventions, namely vitamin D, combined estrogen-progesterone, dehydroepiandrosterone, growth hormone, growth hormone-releasing hormone, combined testosterone-growth hormone, insulin-like growth factor-1, pioglitazone, testosterone, and angiotensin-converting enzyme inhibitors [47]. Recently, new targets and approaches have been identified, and many of them are currently being tested in clinical trials. For example, whether blocking the downstream signaling of IL-6 by the JAK1/2 inhibitor ruxolitinib ameliorates muscle wasting is being investigated in an ongoing study (NCT02072057). Our study has demonstrated that IL-6 and IL-17A were elevated in patients with sarcopenia. Further animal experiments have been proposed to explore the mechanism and investigate the efficacy of monoclonal antibodies in sarcopenia. In addition to drug therapy, lifestyle interventions through diet and exercise seem to be an effective and promising treatment for sarcopenia. Li et al. conducted a study, and they found that nutritional supplementation and resistance exercise can attenuate the subclinical proinflammatory state and ameliorate sarcopenia, as seen by the significant declines in several inflammatory factors [48].

However, there are several limitations to our study. First, it was a single-center retrospective study with a small sample size. Larger prospective studies are necessary to learn more about the association between serum cytokine levels and sarcopenia. Second, the makeup of the sample comprised a greater proportion of men than women. Therefore, there is a risk of selection bias influencing the results and conclusions. The associations demonstrated in our study may be different in a more representative population with an equal proportion of men and women. Third, most aged patients are always present with various chronic diseases. We could not exclude the possibility that these diseases also cause high inflammatory cytokine levels. Whether aging is the direct cause of an increased level of inflammatory cytokines remains unknown, and the molecular mechanism requires further research.

## 5. Conclusions

In conclusion, aging is accompanied by a chronic proinflammatory state. Our research found that in sarcopenic elderly subjects, the serum levels of several biomarkers, such as IL-6, IL-17A, and TNF-*α*, were higher than those in nonsarcopenic elderly persons. Further studies are needed to explore the possible molecular mechanisms and discover new therapeutic targets.

## Figures and Tables

**Table 1:** Characteristics of participants.

Variable	Sarcopenia	Non-sarcopenia	*p* value
Number	35	35	-
Gender, M : F (% men)	21 : 14 (60.0)	22 : 13 (62.9)	0.806
Age, year	75.1 ± 5.16	70.8 ± 5.50	0.001^*∗∗*^
BMI (kg/m^2^)	22.73 ± 2.21	23.81 ± 1.82	0.028^*∗*^
Smoking status			0.762
Never smoker	14 (40%)	16 (45.7%)	
Current smoker	15 (42.9%)	12 (34.3%)	
Ex-smoker	6 (17.1%)	7 (20%)	
Current comorbidities			
Diabetes	10 (28.6%)	8 (22.9%)	0.584
COPD	17 (48.6%)	15 (42.9%)	0.631
ASMI			
Mean	5.64 ± 0.81	6.71 ± 0.73	<0.001^*∗∗∗*^
Men	6.11 ± 0.53	7.25 ± 0.20	<0.001^*∗∗∗*^
Women	4.95 ± 0.65	5.80 ± 0.88	<0.001^*∗∗∗*^
Handgrip strength (kg)			
Mean	21.04 ± 3.88	23.51 ± 5.23	0.028^*∗*^
Men	22.92 ± 3.25	24.83 ± 5.47	0.172
Women	18.21 ± 2.97	21.29 ± 4.08	0.033^*∗*^
Gait speed (m/s)	0.96 ± 0.13	1.15 ± 0.17	<0.001^*∗∗∗*^
SARC-F	6 (5–7)	2 (1–2)	<0.001^*∗∗∗*^
Skinfold thickness (mm)			
Biceps	12 (5–20)	14 (6–28)	0.107
Subscapular	11 (5–22)	14 (5–26)	0.090
Muscle thickness (cm)			
Biceps	1.75 ± 0.17	1.91 ± 0.35	0.019^*∗*^
Quadriceps	2.09 ± 0.29	2.30 ± 0.31	0.004^*∗∗*^
Albumin (g/L)	38.3 ± 2.79	39.5 ± 1.76	0.034^*∗*^

BMI: body mass index; COPD: chronic obstructive pulmonary disease; ASMI: appendicular skeletal muscle index. ^*∗*^*P* < 0.05; ^*∗∗*^*P* < 0.01; ^*∗∗∗*^*P* < 0.001.

**Table 2:** Comparison of levels of inflammatory cytokines.

Variable	Sarcopenia	Non-sarcopenia	*p* value
IL-6 (pg/ml)	14.56 ± 6.62	10.46 ± 4.30	0.003^*∗∗*^
IL-17A (pg/ml)	6.48 ± 2.83	4.97 ± 2.70	0.025^*∗*^
IL-10 (pg/ml)	2.95 ± 1.36	3.86 ± 1.97	0.027^*∗*^
TNF-*α* (pg/ml)	8.01 ± 4.86	5.50 ± 3.72	0.018^*∗*^

IL-6: interleukin-6; IL-17A: interleukin-17A; IL-10: interleukin-10; TNF-*α*: tumor necrosis factor-*α*. ^*∗*^*P* < 0.05; ^*∗∗*^*P* < 0.01.

**Table 3:** Analysis of levels of inflammatory cytokines by gender.

Variable	Males (*n* = 43)			Females (*n* = 27)		
	Sarcopenia (*n* = 21)	Non-sarcopenia (*n* = 22)	*p* value	Sarcopenia (*n* = 14)	Non-sarcopenia (*n* = 13)	*p* value
IL-6 (pg/ml)	15.83 ± 6.79	10.13 ± 4.39	0.002^*∗∗*^	13.95 ± 6.09	10.54 ± 4.26	0.041^*∗*^
IL-17A (pg/ml)	6.03 ± 1.64	4.65 ± 2.43	0.035^*∗*^	7.86 ± 3.33	5.04 ± 2.70	0.023^*∗*^
IL-10 (pg/ml)	3.01 ± 1.49	4.48 ± 1.81	0.006^*∗∗*^	2.86 ± 1.20	3.62 ± 1.83	0.058
TNF-*α* (pg/ml)	8.06 ± 4.46	5.64 ± 2.88	0.040^*∗*^	8.43 ± 3.92	4.24 ± 2.92	0.004^*∗∗*^

IL-6: interleukin-6; IL-17A: interleukin-17A; IL-10: interleukin-10; TNF-*α*: tumor necrosis factor-*α*. ^*∗*^*P* < 0.05; ^*∗∗*^*P* < 0.01.

**Table 4:** Correlation analysis between indexes and sarcopenia.

Variable	*r*	*p* value
Age	0.287	0.016^*∗*^
BMI	−0.146	0.226
Hand strength	−0.391	0.001^*∗∗*^
Gait speed	−0.439	<0.001^*∗∗∗*^
Muscle thickness		
Biceps	−0.253	0.034^*∗*^
Quadriceps	−0.358	0.002^*∗∗*^
Albumin	−0.261	0.029^*∗*^
IL-6	0.265	0.027^*∗*^
IL-17A	0.308	0.009^*∗∗*^
IL-10	−0.301	0.011^*∗*^
TNF-*α*	0.171	0.156

BMI: body mass index; IL-6: interleukin-6; IL-17A: interleukin-17A; IL-10: interleukin-10; TNF-*α*: tumor necrosis factor-*α*; *r*: correlation coefficient. ^*∗*^*P* < 0.05; ^*∗∗*^*P* < 0.01; ^*∗∗∗*^*P* < 0.001.

## Data Availability

The datasets used and/or analysed during the current study are available from the corresponding author on reasonable request.

## Abbreviations

AWGS: Asian Working Group for Sarcopenia
ASM: Appendicular skeletal muscle mass
ASMI: Appendicular skeletal muscle mass index
BIA: Bioelectrical impedance analysis
BMI: Body mass index
COPD: Chronic obstructive pulmonary disease
DXA: Dual-energy X-ray absorptiometry
HS: Handgrip strength
IL: Interleukin
IFN: Interferon
JAK: Janus kinase
MCP: Monocyte chemotactic protein
NF: Nuclear factor
ROR: Retinoic acid-related orphan receptor
ROS: Reactive oxygen species
SD: Standard deviation
STAT: Signal transducer and activator of transcription
TGF-*β*: Transforming growth factor-*β*
TNF: Tumor necrosis factor
US: Ultrasound.
